# Dietary correlates of an at-risk BMI among Inuit adults in the Canadian high arctic: cross-sectional international polar year Inuit health survey, 2007-2008

**DOI:** 10.1186/1475-2891-11-73

**Published:** 2012-09-18

**Authors:** Natalia Zienczuk, T Kue Young, Zhirong R Cao, Grace M Egeland

**Affiliations:** 1 Centre for Indigenous Peoples’ Nutrition and Environment (CINE) and School of Dietetics and Human Nutrition, McGill University, 21,111 Lakeshore Rd, Ste-Anne-de-Bellevue, Quebec H9X 3 V9, Canada; 2 Dalla Lana School of Public Health, University of Toronto, 155 College St., Health Sciences Bldg., Toronto, Canada, M5T 3M7; 3 Norwegian Institute of Public Health and the Department of Public Health and Primary Health Care, University of Bergen, Kalfarveien 31, 5018 Bergen, Norway

**Keywords:** Obesity, High-sugar drinks, Inuit

## Abstract

**Background:**

The study’s objective was to investigate the dietary correlates of an at-risk body mass index (BMI) among Inuit adults from thirty-six communities across the Canadian Arctic using data from the cross-sectional International Polar Year Inuit Health Survey, conducted in 2007–2008.

**Methods:**

The survey included assessments of 24-hr dietary recall, sociodemographics, physical activity, and anthropometry. Dietary characteristics of overweight and obesity were similar and therefore combined into one at- risk BMI category (≥25 kg/m2) for analyses. The relationship between an at-risk BMI and energy intake from macronutrients, high sugar drinks, high-fat foods, saturated fatty acids, and traditional foods were examined entering each dietary variable separately into a logistic regression model as an independent variable. Analyses were adjusted for age, sex, region, kcalories, walking, smoking and alcohol consumption. Further multivariable models considered selected dietary variables together in one model.

**Results:**

An at-risk BMI was present for 64% with a prevalence of overweight and obesity of 28% and 36%, respectively. Consumption of high-sugar drinks (>15.5% E) was significantly related with having an at-risk BMI (OR = 1.6; 95% CI 1.2; 2.2), whereas the % E from total carbohydrate evaluated as a continuous variable and as quartiles was inversely related to an at-risk BMI (*P* -trend < 0.05) in multivariable analyses. While % E from high-fat foods was positively related to an at-risk BMI, the findings were not significant in a model controlling for high-sugar drinks and % E from carbohydrates.

**Conclusions:**

The prevalence of overweight and obesity is of public health concern among Inuit. The current findings highlight the obesogenic potential of high-sugar drink consumption in an ethnically distinct population undergoing rapid cultural changes and raises concerns regarding carbohydrate restricted diets. Health promotion programs aimed at preventing the development of an unhealthy body weight should focus on physical activity and the promotion of healthy diets with reduced intake of sugar drinks.

## Background

Inuit have undergone rapid changes affecting all dimensions of life with implications for nutrition and epidemiologic transitions. Obesity has been declared a global problem and the Arctic is no exception [1-3]. In Canada, the BMI-based prevalence of obesity was 23% while the prevalence among the off-reserve Canadian Aboriginal population was nearly double that amount [4]. Age-standardized BMI data indicated that Inuit populations have a high prevalence of obesity compared to other populations in Europe and North America [5]. Pooled data from 4 separate studies on Inuit anthropometry conducted between 1990 and 2000 found that Inuit men ranked in the upper half of 76 countries while Inuit women ranked in the top quintile of 115 countries when crude prevalence data from other countries published by the International Obesity Task Force was used for comparison [5].

Obesity, especially abdominal obesity, is an independent risk factor for a number of co- morbidities and chronic illnesses, including cardiovascular disease, type 2 diabetes mellitus, as well as a cluster of metabolic disturbances [2,4-7]. Inuit, a term used to collectively include Inuit, Inupiaq, Yupik, and Kalaallit, have historically been free of such chronic diseases, which are now known as ‘diseases of modernization’ [8]. Changing dietary patterns with greater reliance upon store bought food versus traditional food are believed to contribute to the burden of overweight and obesity and obesity-related chronic diseases [9,10]. A study on dietary change among Indigenous Peoples of the Canadian Arctic revealed only 10-36% of adult dietary energy was derived from traditional food [9].

However, there is a lack of nutritional epidemiological data outlining the dietary associates of overweight and obesity among Indigenous Peoples undergoing rapid changes. Studies evaluating the dietary correlates of overweight and obesity are important for providing a context for understanding and preventing the obesity epidemic. The objective of the current study was to evaluate the dietary factors associated with overweight and obesity among Inuit adults in northern Canada using the cross-sectional International Polar Year Inuit Health Survey, 2007–2008.

## Methods

### Study population and location

Details of the study population and methodology have been presented elsewhere [2,3]. The study population consisted of Inuit adults aged 18 years or older who resided in northern Canada and were included in the International Polar Year Inuit Health Survey in 2007 and 2008. The survey took place in the late summer and fall of 2007 and 2008 in 33 coastal communities through the use of the Canadian Coast Guard Ship Amundsen, and in 3 non-coastal communities using land teams. The communities included all communities in three regions of northern Canada: Nunavut, Nunatsiavut, and the Inuvialuit Settlement Region of the Northwest Territories. Homes were assigned numbers and randomly selected using a computerized random digit assignment or table. Inuit adults in randomly selected homes were invited to participate. Pregnant women were not eligible to participate. Research personnel included trained bilingual interviewers, community research assistants, nurses, lab and other technicians, and quality control staff. Consent forms, an informative DVD, and all questionnaires were in Inuit languages and dialects and English. The study was developed in a participatory approach with members of the steering committees of 3 jurisdictions: Inuvialuit Settlement Region, Nunavut Territory, and Nunatsiavut Region, the new land claim area in northern Labrador. The McGill Faculty of Medicine Institutional Review Board issued a certificate of Ethical Acceptability. Informed consent was obtained from each participant.

### Questionnaires

The cross-sectional study for adults consisted of interviewer-administered questionnaires. The International Physical Activity Questionnaire (IPAQ) short form was used to assess participants’ physical activity during the previous seven days [11] in which metabolic equivalent (MET) intensity levels were taken from the comprehensive compendium of physical acivity [12]. In an earlier analyses of the current study population, walking MET scores were found to be significantly associated with an at-risk BMI whereas MET scores for moderate and vigorous-intensity activity were not found to be significantly related with an at-risk BMI [3] possibly reflecting inadequacies of the short form 7-day IPAQ for capturing habitual intake or participant reporting errors. IPAQ short form was chosen for the current study due to community concerns regarding research burden to participants.

Interviewers were trained in administering the 24-hour recall based on an adaptation of 5-stage multiple-pass recall interviewing technique developed by the United States Department of Agriculture (USDA) designed to maximize an individual’s recollection of foods consumed the previous day, beginning at midnight (00:01) and ending at midnight (24:00) [13,14].The recall queries involved a quick list, time and occasion, forgotten foods and a detailed cycle review. Description of amounts and portion sizes were aided by portion size model kits (Santé Québec). Dietary recall data were entered using CANDAT Software (Godin London) and nutrient composition of foods was determined using the 2007b Canadian Nutrient File [15] with supplemental information on nutrient composition from an in house (School of Dietetics and Human Nutrition) food file for foods not represented in the Canadian Nutrient File [13]. Data entry of recalls was double verified. When recalls did not include recipes for mixed dishes, northern recipe default values obtained through regional contacts and detailed dietary recalls were used. A total of 74 dietary recalls were invalidated due to either insufficient information (n = 28); participant deciding to not continue with the recall (n = 17); or misunderstanding regarding the length of fasting (n = 29) (i.e., participants were requested to fast for eight hours).

Intakes of nutrients and total energy were estimated using data collected by the 24 hour recall. Due to logistical constraints a repeat dietary recall was not collected. As food frequency questionnaire data were available for past-year traditional food intake and past-month beverage consumption, we evaluated correlations between the 24-hr traditional food and high-sugar drink data with that of the FFQ data. The correlation coefficient comparing high-sugar drink consumption noted in the 24-hr recall and the past-month beverage FFQ was statistically significantly related with an *r* of 0.45, *P* < 0.0001. For traditional food consumption, the correlation coefficient between the past 24-hr and the past-year FFQ intake data was 0.30 (*P* < 0.001).

### Physical measurements

Trained nurses measured weight using a Tanita instrument (Tanita Corporation Tokyo, Japan) and height using a stadiometer. Participants were asked to remove shoes and socks and a standard clothing weight was subtracted (0.4 kg) for all participants.

### Coding of variables

Walking was based on calculated MET scores using an energy requirement of 3.3 and the continuous variable used in analyses. Obesity was defined as BMI ≥ 30 and overweight as BMI ≥ 25 and < 30 kg/m^2^[16] and an at-risk BMI was defined as BMI ≥ 25 kg/m2.

Dietary factors were evaluated as nutrient densities (nutrient in kcalories/total kcalories) and total kilocalories was also entered as an additional covariate. High-sugar drinks were defined as sodas, juices, sports drinks, and punches containing >25% of total energy as sugar and excluded diet sodas and real fruit juices. High-sugar drink intake was not normally distributed with approximately 30% consuming no high-sugar beverages, thus high-sugar drink consumption was divided into three categories based on approximate tertiles: non-consumer, low (1–15.5% of E), and high (>15.5% of E) consumer, where non-consumers were used as the reference category. Likewise, a high percent consumed no soft drinks, thus this variable was evaluated as percent soft drink consumers (yes vs no) in which diet soda consumers were excluded.

High-fat foods were defined as foods containing >40% of total energy as fat and included both market food and traditional food sources of fat. The traditional food variable was composed of traditional foods from the predominantly meat-based Inuit diet, which included various sea mammals, land animals, fish, birds, and plants and excluded any beverages or bannock. Percent of total energy intake from saturated fatty acids (SFA) included both traditional and market food sources of saturated fatty acids. To calculate the potential underreporting of dietary intake, the ratio of reported energy intake (EI) to basal metabolic rate (BMR) was calculated using the FAO/WHO/UNU equation and a value below 1.5 was used to identify underreporting [17].

A Canadian version of the USDA Healthy Eating Index (HEI) was used in this study based on Canadian age and sex specific dietary recommendations (maximum score = 100). HEI scores were calculated in this study and compared among overweight/obese versus normal weight individuals by gender. The HEI assesses how diet adheres to dietary recommendations in which the Canadian modifications are based upon Canada’s Food Guide to Healthy Eating and the Nutrition Recommendations for Canadians. The Canadian HEI has nine sub scores each worth a maximum of 10 points, with the exception of the fruits and vegetables sub score which is worth 20 points, for a total maximum of 100 points. The Canadian HEI measures diet quality by assessing number of servings from the main food groups, intake of dietary fats, sodium, as well as diet variety [18].

### Statistical analyses

Socioeconomic (SES) characteristics were similar between overweight and obese participants [3]. Also, dietary characteristics did not differ between overweight and obese individuals in multivariable logistic regression. Thus, the overweight and obese groups were combined into one at-risk category for analyses. Logistic regression analyses were performed using at-risk BMI (BMI > =25 kg/m^2^ ) as the dependent variable and each nutrient was evaluated separately in models as the independent variable. With the exception of fiber and HEI, all models used the nutrient density approach (% E); total kilocalories was entered as a covariate. In the evaluation of dietary associations with an at-risk BMI, we did not control for SES indicators because SES is a strong determinant of diet quality and one means by which SES may contribute to obesity. Dietary analyses adjusted for age, sex, survey region (Nunavut, Nunatsiavut Region, and Inuvialuit Settlement Region), walking (continuous IPAQ MET score), current smoking, and past-year alcohol consumption (yes vs no). Additional multivariable modeling adjusted for the aforementioned variables in addition to important nutrients. Cluster analyses were used for logistic regression modeling since there were, on average, 1.38 participants per home. All statistical analyses were performed using STATA version 10.0 statistical software package. Statistical significance was declared at *P* < 0.05.

## Results

A total of 2,796 households were approached of which 1,901 (68%) agreed to participate with a total of 2,595 participants of whom 998 were male and 1597 were female. Body mass index was available for 84% (n = 2,178) of study participants and a valid dietary recall was available for 81% (n = 2097) of study participants. With 28% overweight and 36% obese, an at-risk BMI was present for 64% of participants (Table 1). Current smoking was highly prevalent (70%) as was any alcohol consumption in the past-year (66%), and residence in public housing (64%).

**Table 1:** Demographic characteristics of study population: international polar year inuit health survey, 2007-2008

**Characteristics**	**N**	**%**
Female	2595	61.5
BMI Measures	2178	27.9
Overweight		
Obese		35.8
Current smoking	2206	69.8
Alcohol in past year	1888	66.3
High-school or greater education	2174	38.4
Living in public housing	2537	64.1
Age	2595	
<30 yrs		23.6
30 ~ 40 yrs		26.0
> 40 yrs		50.4

Sixty percent of men and 66% of women had an at-risk BMI, with women having a higher prevalence of obesity than men (42% and 27%, respectively), and men having a higher prevalence of overweight than women (33% and 25%, respectively). The prevalence of an at-risk BMI decreased with increasing tertile category of walking (58%, 63%, and 69% in high, medium, and low tertiles of walking; *P* < 0.05; *X*^2^ test for trend *P* < 0.01).

### Energy intake from 24-hour recall comparing those with and without an At-risk BMI

In multivariable regression analyses no dietary differences emerged between overweight and obese individuals; thus, overweight and obese individuals were combined into one at-risk category for further multivariable logistic regression analyses. Also, in analyses of dietary intake data, adjusted for age and energy intake and stratified by sex, similar patterns in dietary intakes and similar energy intake were noted for men and women when comparing those with and without an at-risk BMI (data not shown). However, the EI:BMR values indicated that men under-reported energy intake regardless of weight status whereas women with an at-risk BMI underreported energy intake to a greater degree than women without an at-risk BMI (for men EI:BMR of 1.4 regardless of weight status, for women EI:BMR of 1.3 and 1.7 for those with at-risk BMI and healthy BMI, respectively, *P* < 0.001).

### Dietary factors associated with an at-risk BMI

In multivariable analyses, adjusting for age, sex, region, walking, total kilocalories, current smoking and alcohol consumption, the percent of E from carbohydrates had a mild inverse relationship with an at-risk BMI (*P* ≤0.10), whereas high-sugar drinks in the second (1%-15.5% of E) and third tertile (>15.5% E) relative to the lowest tertile of non-consumers, was associated with an increased odds of an at-risk BMI (OR 1.4, 95% CI 1.1, 2.0 and OR 1.6, 95% CI 1.2-2.2, for second and third tertiles, respectively (Model 1, Table 2). No other nutrients were significant in model 1 analyses. In a multivariable model simultaneously considering % E carbohydrates, high-sugar drink tertiles and % E from high-fat foods, the significant associations of high-sugar drink consumption and an at-risk BMI persisted and % E carbohydrate was inversely related to an at-risk BMI (*P* < 0.01) (Model 2, Table 2). Further consideration of percent of energy from traditional food in additional multivariable models did not identify any significant associations with an at-risk BMI and did not alter the *beta* coefficients associated with the other dietary variables.

**Table 2:** Adjusted logistic regression coefficients and odds ratios (OR) for each diet quality indicator separately evaluated for predicting an at-risk BMI (> = 25 kg/m2) after adjustment for potential confounding factors in inuit adults

**Independent variables**	**Model 1**^**1**^		**Model 2**^**2**^	
	**OR (95%CI)**	**p-value**	**OR (95% CI)**	**p-value**
**% E Protein**	1.01(0.99-1.02)	0.31		
**% E Fat**	1.00(0.99-1.01)	0.81		
**% E Carbohydrates**	0.99(0.99-1.00)	0.09	0.99(0.98-1.00)	<0.01**
**Fibre (g)**^**3**^	0.99(0.97-1.01)	0.33		
**% E High Sugar Drinks**^**4**^				
Low (1%-15.5%) vs No	1.43(1.05-1.95)	0.02*	1.53(1.12-2.11)	<0.01**
High (>15.5%) vs No	1.59(1.15-2.19)	<0.01**	1.91(1.34-2.72)	<0.01**
**% Soft Drinks**				
Yes vs No	1.25(0.96-1.61)	0.10		
**% E High Fat Foods**^**5**^	1.00(1.00-1.01)	0.28	1.00(0.99-1.01)	0.84
**% E Saturated Fat**	1.00(0.97-1.03)	0.83		
**% E Traditional Food**				
Low (1% - 25%) vs No	1.00(0.75-1.33)	0.98		
High (26%-100%) vs No	1.16(0.84-1.62)	0.37		
**HEI**^**6**^	1.00(0.99-1.01)	0.77		

*Note*. Dietary factors are evaluated as a % of total energy intake and adjusted for caloric intake in regression analyses with the addition of total kilocalories as an additional covariate.

^1^Adjusted for age (years), sex, region (ISR, Nunavut, Nunatsiavut), and walking (continuous MET score), total kilocalories, current smoking and drinking in past the year.

^2^Adjusted for age (years), sex, region, walking (continuous MET score), total kilocalories, % E from carbohydrates, % E from high sugar drinks, % E from high fat foods, current smoking and drinking in past the year. Not adjusted for % E from carbohydrates for the % E carbohydrates model, not adjusted for % E from high sugar drinks for the high sugar drinks model, not adjusted for % E from high fat foods for the % E from high fat foods model.

^3^Total kilocalories entered as an additional covariate.

^4^Percent of total energy from drinks with >25% of energy as sugar.

^5^Percent of total energy from foods with >40% energy as fat.

^6^Based on the Canadian Food Guide, HEI assigns a score (max = 100) based on diet quality of the individual; total kilocalories entered as an additional covariate.

* *P* < 0.05; ** *P* < 0.01.

In additional analyses evaluating quartile groupings of intake of percent E from carbohydrates and high-fat foods, a significant trend with decreasing prevalence of an at-risk BMI was observed across increasing quartiles of carbohydrate intake (70%, 66%, 63%, and 57%, respectively, test for trend *P* < 0.05), but not for increasing quartiles of high-fat food intake (test for trend p = 0.94) in multivariable analyses.

As dietary exposures are complex and inter-related, diet quality indicators were evaluated by carbohydrate intake quartile groupings, in which % E from protein, fat, and saturated fat were significantly inversely related to carbohydrate exposures, while fiber intake was positively related to carbohydrate intake (Table 3).

**Table 3:** Mean (SD) population characteristics by quartile of carbohydrate intake evaluated as a percent of total energy intake

	**Quartile 1 (Median = 26.0%)**		**Quartile 2 (Median = 41.5%)**		**Quartile 3 (Median = 52.5%)**		**Quartile 4 (Median = 12.4%)**			
	**Mean**	**SD**	**Mean**	**SD**	**Mean**	**SD**	**Mean**	**SD**		
Age	48.2	15.1	42.4	14.6	39.8	14.1	35.7	12.4 ***
HEI^1^	45.6	11.5	51.9	11.6	57.7	11.4	58.0	10.7 **
Total Energy (kJ)	9467.1	6072.7	9405.6	4992.8	9311.5	4669.8	7970.9	4875.2***
% E Protein	31.4	13.2	22.1	7.99	17.3	5.90	12.1	5.26***
% E Fat	40.0	13.5	34.7	8.48	30.1	6.58	21.2	7.22 ***
% E Traditional Food	38.2	27.4	16.0	16.9	8.46	10.9	4.81	7.76 ***
% E SFA	12.0	5.09	11.6	4.57	10.2	3.00	7.43	3.14 **
Total Fibre (g)	6.17	6.34	10.0	7.99	11.7	8.19	9.67	8.30***
Fruit/Veg Fibre (g)	3.83	1.34	3.82	1.10	3.80	1.03	3.50	1.08 ***
Cold Cereal Fibre (g)	0.45	0.20	0.45	0.17	0.45	0.15	0.40	0.16 ***
Hot Cereal Fibre (g)	0.14	0.04	0.14	0.03	0.13	0.03	0.13	0.03
Mixed Grain Fiber^2^ (g)	0.78	0.37	0.78	0.31	0.77	0.29	0.69	0.30 ***
Pasta Fibre (g)	0.62	0.33	0.62	0.27	0.61	0.25	0.54	0.26 ***
Walking METs	1510.3	1552.1	1244.0	1368.0	1212.9	1393.3	1435.6	1509.2

*Note.* Dietary factors are adjusted for total energy using the nutrient density approach with the

exception of fibre where total kilocalories were entered as an additional covariate.

1Based on the Canadian Food Guide, HEI assigns a score (max = 100) based on diet quality of the individual.

2Mixed Grain Fibre variable includes fibre in grams from mixed foods including grains.

* *P-*trend <0.05; ** *P* < 0.01; *** *P* < 0.001.

### Healthy eating index and obesity

The nine subscores of the Canadian HEI were also examined in relation to overweight/obesity in men and women separately but no significant associations between HEI subscores and an at-risk BMI were observed. The mean HEI score was 53.3 (SD =12.4) and ranged from 12.6 to 96.2. Women had a slightly higher mean HEI than men (55.4, SD = 12.5 versus 49.9, SD = 11.5, respectively; *P* < 0.01). No significant associations were observed between HEI score and an at-risk BMI in logistic regression analysis (Table 2). Increasing carbohydrate quartiles were associated with increasing HEI scores (Table 3).

## Discussion

### Prevalence of obesity

The Inuit Health Survey 2007–2008 identified a level of overweight and obesity among Inuit adults that is slightly higher than that observed in the 2004 Canadian Community Health Survey [19] and of that reported from 4 circumpolar Inuit populations in the 1990’s [20]. According to our survey data, 60% of Inuit men and 66% of Inuit women were either overweight or obese indicating that prevention efforts are needed.

Our study found that high-sugar drink consumption was positively associated with an at-risk BMI while total carbohydrate intake, adjusting for sugar-drink consumption, was inversely related to an at-risk BMI. Our findings are similar to that observed among Cree of Northern Québec, where high-sugar drink consumption was positively associated and total carbohydrate intake was inversely associated with BMI [21].

Our findings that high-sugar drink intake was associated with increased odds of an at-risk BMI is meaningful in that it adds to the literature regarding the obesogenic potential of high-sugar drink intake in adults in an ethnically distinct population. While the literature is inconsistent regarding the association of high-sugar drink intake and obesity among adults, [22] evidence suggests that carbohydrates in liquid form may not have the same effect on satiety as that provided by food intake which over the long-term would theoretically result in excess energy intake and resulting weight gain [23].

The strong inverse relationship between carbohydrate intake and having an at-risk BMI raises concerns regarding diets which overly restrict carbohydrate intake. The median % energy from carbohydrate in the highest quartile (65.3%) is comparable to the high-end of the Acceptable Macronutrient Distribution Range (AMDR) for carbohydrate intake of 65%, whereas the lowest quartile median percent energy from carbohydrate (26%) was below the low-end of the AMDR for carbohydrate intake of 45%. AMDRs are a range of macronutrient intakes associated “with reduced risk of chronic disease while providing adequate intakes of essential nutrients” [24]. Therefore, the mean carbohydrate intakes are quite low in the study population.

In support of our findings that carbohydrate intake was associated with decreased odds of an at- risk BMI, sustained weight loss in individuals on low carbohydrate diets have been documented for a duration of 6 months but few studies have found these diets to be successful for longer than a year [25]. The long-term success of low carbohydrate diets is controversial and most of the short-term benefits are attributable to total body water loss and decreased calorie intake due to elimination of most foods and food variety in the diet [26]. Furthermore, few longitudinal studies on low carbohydrate diets have been conducted for periods of greater than 12 months due to high drop-out rates because of difficulty adhering to such diet regimens [27]. Low carbohydrate diets eliminate important foods such as fruits and vegetables high in vitamins and minerals and often have no restriction on saturated fat intake – all of which are dietary intake patterns associated with chronic disease. Our results support the hypothesis that the association between carbohydrates and overweight is dependent on the quality of the carbohydrate since we found that carbohydrates were negatively associated with an at-risk BMI while high-sugar drink intake was associated with increased odds of overweight and obesity. While carbohydrates can be classified based on their glycemic index, [28] the Canadian Nutrient File does not contain glycemic index of foods and the glycemic potential of carbohydrates can be modified by protein and fat in meals. The carbohydrates with a low glycemic index include slowly digestible and resistant starches which are considered beneficial in that they promote satiety and prevent the reactive hypoglycemia associated with sugars [23,29-31]. In our analyses, total fibre intake was not significantly associated with odds of an at-risk BMI, however total fibre intake was very low in the current study population with a mean of 9.8 g (SD = 8.0 g) compared to recommended intake of 38 g and 25 g for men and women under 50 years, respectively [24].

Similar to our findings, no significant differences in traditional food consumption was observed by BMI category in an earlier study of Yukon First Nations, Dene/Metis, and Inuit communities and a Greenlandic study [9,32]. Recent studies among Inuit adults in Arctic Canada reported the majority of participants were classified as overweight and obese, yet also reported medium to high physical activity levels [33].

Overall, the HEI scores among Inuit were suboptimal and indicative of a poor quality diet as is also the case for Canadians based on the Canadian Community Health Survey (CCHS) 2004 cycle where only 0.5% of the population had an HEI score of more than 80 [34].

## Limitations

Limitations of our particular study include that dietary information was collected using a single 24-hour recall, which is practical for population level studies but makes it difficult to portray dietary patterns for smaller subgroup analyses. However, the logistics of the field survey work precluded the incorporation of a repeat dietary recall or lengthy FFQs. Also, the survey was conducted in one season (late summer/early fall). Though seasonality could be an important factor that may affect the balance of market to traditional food consumption, previous studies have documented that market food intake is remarkably similar between two seasons: 69.8% of total energy intake by Dene/Metis in the summer versus 63.4% of total energy intake in the winter [35]. Further, market food intake was shown to be consistent among Dene/Metis and Yukon children both with and without traditional food consumption, especially regarding market food items that fall into the “other” food group (sweets, fats, mixed dishes, etc.) which accounted for more than 40% of total energy derived from market food [36]. Another limitation is that the calculated EI:BMR ratios for assessment of level of underreporting in this study were suboptimal, with greater under-reporting among those with an at-risk BMI, illustrating the difficulties of evaluating energy intake as a key determinant of an at-risk BMI. However, evaluation of energy adjusted nutrient intakes allows us to overcome the bias associated with under-reporting of EI assuming that under-reporting is not specific to any nutrient or food item. Furthermore, as the present study was a cross-sectional study a potential limitation is the possibility of reverse causality where lower carbohydrate intake among those with an at-risk BMI may simply reflect the popularity of low carbohydrate diets for weight loss. However, reverse causality for carbohydrate intake is unlikely given the observed association between high-sugar drinks and an at-risk BMI.

Finally, another limitation is the men were under-represented in the survey which may have resulted in biased estimates of population characteristics of men.

## Conclusion

Evaluating the correlates of obesity among Inuit provides a context for understanding potential contributing factors and will help tailor health promotion programs aimed at preventing the development of obesity. Encouraging walking and other activities, increased fruit and vegetable consumption, advocating increased consumption of traditional food sources of fibre, promotion of dietary balance in macronutrient intake, and a reduction of high-sugar drink intake may be effective strategies for combating the obesity epidemic in the Canadian Arctic.

## Abbreviations

BMI: Body mass index; BMR: Basal metabolic rate; EI: Energy intake; FFQ: Food frequency questionnaire; HEI: Healthy eating index; IPAQ: International physical activity questionnaire; MET: Metabolic equivalents; SES: Socioeconomic status; SFA: Saturated fatty acids; USD: United states department of agriculture.

## Competing interests

The authors declare that they have no competing interests.

## Authors’ contributions

NZ and ZC analyzed the research data, NZ interpreted the findings and wrote the manuscript. GE and TKY designed the study and supervised data collection. GE guided the statistical analyses and both GE and TKY facilitated interpretation of the results and edited the manuscript. All authors read and approved the final manuscript.
